# An ideal portrait of the professional competence of clinical research nurses: A qualitative study

**DOI:** 10.1016/j.apjon.2025.100682

**Published:** 2025-03-08

**Authors:** Heng Yang, Yipei Chen, Xin Peng

**Affiliations:** a Nursing Department, Union Hospital of Tongji Medical College of Huazhong University of Science and Technology, Wuhan, China; b School of Nursing, Huazhong University of Science and Technology, Wuhan, China

**Keywords:** Nurses, Clinical trial, Professional competence, Qualitative research

## Abstract

**Objective:**

This study aims to identify and define the ideal professional competencies of Clinical Research Nurses (CRNs) in China, focusing on the essential knowledge, skills, and personal attributes required for effective practice in clinical trials.

**Methods:**

Interviews were conducted with CRNs, Nurse Managers (NMs), Principal Investigators (PIs), Sub-Investigators (SIs), Clinical Research Coordinators (CRCs), Clinical Research Associates (CRAs), and subjects. Thematic analysis was performed using Colaizzi's seven-step method to analyze interview data and identify key competencies.

**Results:**

The study identified four primary themes that characterize the ideal CRN profile: (1) theoretical knowledge ability, (2) practical technical ability, (3) professional quality and ability, and (4) personal traits. A total of 21 specific indicators were delineated, reflecting the diverse expectations of various stakeholders in clinical trials.

**Conclusions:**

The findings highlight the multifaceted nature of CRN competencies, emphasizing the importance of comprehensive training programs tailored to the needs of CRNs. This study provides a foundational framework for enhancing CRN training and professional development in China.

## Introduction

A Clinical Research Nurse (CRN) is a professional responsible for conducting research-related tasks that do not require independent medical decision-making in clinical trials, following relevant training and authorization by the principal investigator.^1^^,^^2^ In 2023, the total number of registered clinical trials on the China Drug Clinical Trial Registration and Information Disclosure Platform surpassed 3,410, marking the highest number of registrations in recent years.^3^ Alongside the rapid growth and expansion of clinical trials, the CRN workforce has also been expanding. CRNs play a vital role in coordinating and executing clinical trials.^4^ The professionalization of CRNs has thus emerged as a critical trend in the evolution of the nursing profession.^5^ Despite growing opportunities, CRN development in China remains in its early stages, with competencies yet to be fully defined.^6^ Through descriptive qualitative research, this study aims to outline the ideal profile of CRN professional competencies from a multidisciplinary clinical trial team perspective, contributing to the exploration and establishment of a training system tailored to China's specific needs.

The role of CRNs has become increasingly vital in the landscape of clinical trials, particularly in the context of China's growing health care and pharmaceutical sectors.^7^^,^^8^ These professionals play a crucial role in ensuring the integrity and efficiency of clinical research processes, bridging the gap between clinical care and research objectives.^9^

Despite the growing importance of CRNs, their professional capabilities and competencies have not been comprehensively defined or standardized.^10^ The development of CRNs in China is still in its nascent stages, with many practitioners navigating the complexities of their role without a clear framework for professional growth.^11^ This gap highlights the need to explore and articulate the essential skills and knowledge that CRNs should possess, ensuring they can effectively manage clinical trials and advocate for the rights and well-being of trial subjects.^12^^,^^13^

Furthermore, as the clinical trial landscape evolves, CRNs must adapt to new regulations, technological advancements, and the increasing complexity of trials.^14^ This necessitates a robust understanding of theoretical knowledge, practical skills, professional qualities, and personal traits that define successful CRNs. By identifying and refining these competencies, health care institutions can enhance CRN training programs and create a supportive environment for professional development.^15^

In light of these considerations, this study aims to delineate the ideal profile of CRN professional abilities through qualitative research. By engaging with various stakeholders in the clinical trial process, the research seeks to provide actionable insights that can inform training and development initiatives, ultimately enhancing the effectiveness of CRNs in their critical role.

## Methods

### Study design and the participants

A descriptive qualitative study was conducted using purposive sampling to select four tertiary grade A hospitals with clinical trial qualifications in Hubei Province. Tertiary public hospitals are the main institutions that undertake clinical trials in China. They usually have a complete clinical research system and are equipped with experienced clinical research teams, and the professional ability of CRNs is highly required. Therefore, studying CRNs in these hospitals helps to identify more comprehensive and high standard competency characteristics. As clinical trials have strict GCP compliance requirements, the experience of CRNs in these hospitals can provide a reference for CRN training and competency assessment nationwide. To comprehensively define the professional competencies of CRNs, this study included interviews with not only CRNs but also other key stakeholders involved in clinical trials, including Nurse Managers (NMs), Principal Investigators (PIs)/Sub-Investigators (SIs), Clinical Research Coordinators (CRCs), Clinical Research Associates (CRAs), and trial subjects. Each of these roles interacts with CRNs in different capacities and provides valuable insights into the expectations and competencies required for effective CRN performance. (1) NMs: Supervise CRNs, provide professional development guidance, and assess their competency and performance in research settings. (2) PIs/SIs: Oversee the entire clinical trial, ensuring protocol adherence and regulatory compliance. CRNs work closely with them, supporting trial execution and patient management. (3) CRCs: Assist in managing research logistics, data collection, and subject follow-up. Their collaboration with CRNs helps ensure smooth trial operations. (4) CRAs: Represent sponsors or contract research organizations (CROs) and monitor trial compliance. Their perspective provides insights into CRNs' role in ensuring high-quality clinical trial conduct. (5) Trial Subjects: Their experiences with CRNs contribute to understanding the patient-centered skills required, such as communication, ethical conduct, and informed consent management. CRNs typically report to NMs or PIs, depending on institutional structure. While they primarily work under the PI's supervision regarding trial-related tasks, their nursing responsibilities and professional development are often overseen by NMs. Understanding the expectations of both groups helps in defining the competencies necessary for CRNs to navigate their dual roles in research and clinical care. To ensure privacy, participants were assigned letters based on the order of their interviews: CRN (A), NM (B), PIs/SI (C), CRA (D), CRC (E), and Trail Subject (F). All participants consented to the interviews and agreed to the recording or documentation of the sessions. The sample size was determined based on data saturation, with no new themes emerging from further interviews.

### Terminology clarification

In this study, the term “participants” refers to individuals who took part in the qualitative interviews, including CRNs, NMs, PIs/SIs, CRCs, CRAs, and trial subjects. Among them, “trial subjects” specifically refers to individuals who were enrolled in clinical trials as study participants.

### Inclusion and/or exclusion criteria

CRN inclusion criteria: (1) clinical nursing work for more than 2 years; (2) obtained national GCP (Good Clinical Practice, Good Clinical Practice for drugs and/or devices) training certificate; (3) participating in ≥ 3 drug clinical trials. Exclusion criteria: (1) absence from the post for more than 3 months in the past year for reasons such as cumulative vacation or going out for further study; (2) individuals who have not participated in a complete clinical trial were excluded.

NM inclusion criteria: (1) nursing management work ≥ 5 years; (2) obtain GCP certificate; (3) participated in drug clinical trials for more than 5 years or more than 10 clinical trials. Exclusion criteria: same as above.

PI/SI inclusion criteria: (1) obtaining GCP certificate; (2) associate senior title or above; (3) participated in drug clinical trials for more than 5 years or more than 10 clinical trials. Exclusion criteria: same as above.

CRA and CRC inclusion criteria: (1) obtaining GCP certificate; (2) participating in drug clinical trials for ≥ 2 years; (3) bachelor degree or above. Exclusion criteria: same as above.

Subject inclusion criteria: (1) no communication disorder; (2) without mental disorder; (3) age ≥ 18 years old.

### Data collection

Through literature review and focusing on CRN professional ability, the interview outlines for CRN, NM, PI/SI, CRC, CRA and subjects were developed respectively. According to the results of a small scale pre-interview, the understanding ability of the respondents and the feasibility of the interview purpose, the interview outlines for six groups of people were finally formed (Table 1). Ten participants were interviewed by telephone, while the remaining 30 participants took part in one-on-one, face-to-face interviews. Telephone interviews were scheduled at a mutually convenient time, ensuring a quiet environment for the conversation. Face-to-face interviews were held in a quiet, private office to avoid any disturbances. Before each formal interview, participants introduced themselves, and the purpose and significance of the study were explained. Participants were assured that the interview content would be kept confidential. Informed consent was obtained, and consent forms were signed. Throughout the formal interviews, synchronous recording was used, while avoiding attention to facial expressions and body movements. Each interview lasted no more than 45 minutes, and interviews were concluded when no new information emerged from the data. The data collection period was from August 1 to August 15, 2024.

**Table 1:** Interview outlines.

Interview outlines	
CRN	1. What types of clinical research projects do you often work on? What specialized skills and knowledge are required for these programs?
	2. What challenges do you often have to solve? How do you address these challenges?
	3. What roles and responsibilities do you think clinical research nurses have?
	4. What skills and traits do you think a good clinical research nurse should possess?
NM, PI/SI	1. What skills do you think clinical research nurses need to have?
	2. Can you give me an example of the difficulties encountered in managing clinical research nurses?
	3. What do you think are the roles and responsibilities of clinical research nurses?
	4. What skills and traits do you think a good clinical research nurse should possess?
	5. How do you evaluate the quality of work of clinical research nurses?
CRC, CRA	1. What are the specific work contents of clinical research nurses that you come into contact with in your daily work?
	2. Can you describe in detail one of your most impressive experiences while working with clinical research nurses?
	3. What skills do you think clinical research nurses need to continuously learn in the evolving drug clinical trials?
	4. What role do you think clinical research nurses play in the advancement of clinical trials?
Subject	1. What services have clinical research nurses provided for you since you accepted the drug clinical trial program?
	2. What abilities and qualities do you think nurses need to have in order to better serve the subjects?
	3. What do you think needs to be improved in the work of clinical research nurses?
	4. What was the most satisfying and dissatisfying thing about your nursing work since you accepted the drug clinical trial program? Could you tell me about the situation and your thoughts?

CRN, Clinical Research Nurse; NM, Nurse Manager; PI, Principal Investigator; SI, Sub-Investigators; CRC, Clinical Research Coordinator; CRA, Clinical Research Associates.

### Data analysis

Within 24 hours after each interview, two researchers repeatedly listened to the recordings and transcribed them, incorporating notes taken during the interviews. The written data were then compared and analyzed. Any discrepancies were identified and clarified by contacting the respondents to reach a consensus. Interview data were analyzed using Colaizzi's seven-step^16^^,^^17^ method to ensure a systematic and rigorous approach: Step 1: Familiarization with Data: Two researchers independently transcribed and repeatedly read through the interview transcripts to gain an in-depth understanding of the content. Step 2: Identification of Significant Statements: Meaningful statements related to CRN competencies were extracted verbatim from the transcripts. Step 3: Formulating Meanings: Each significant statement was assigned a preliminary code that captured its meaning. Step 4: Clustering into Categories: Similar codes were grouped into subcategories, which were refined and reviewed iteratively to form emerging patterns. Step 5: Defining Themes: Subcategories were synthesized into broader themes representing key aspects of CRN professional competence. Step 6: Theme Extraction and Bias Reduction: To minimize researcher bias, two researchers independently extracted and coded themes before comparing their findings. Any discrepancies were resolved through team discussions until consensus was reached. All themes were cross-checked with original transcripts to ensure they accurately reflected participants' perspectives. Step 7: Validation with Participants: The final themes were returned to participants for verification using open-ended, non-leading questions (e.g., “Do these themes reflect your experience?”). Their feedback was documented verbatim and incorporated without modification to maintain authenticity and avoid researcher influence. An example of the coding process can be found in the Supplementary materials.

### Reporting method

The Consolidated criteria for reporting qualitative research (COREQ) checklist was used when reporting the results.^18^

## Results

### Participant characteristics

A total of 10 CRNs, 5 NMs, 5 PI/SIs, 10 CRAs, 5 CRCs, and 5 subjects were included. Their general characteristics are shown in Table 2, Table 3.

**Table 2:** Characteristics of CRNs, NMs and PI/SIs (*N* ​= ​20).

	CRN (*n* ​= ​10) *n* (%)	NM (*n* ​= ​5) *n* (%)	PI/SI (*n* ​= ​5) *n* (%)
**Sex**			
Male	0	0	2 (40)
Female	10 (100)	5 (100)	3 (60)
**Age(years,mean ± SD)**	27.00 ​± ​2.98	37.80 ​± ​2.59	40.20 ​± ​7.98
**Professional title**			
Nurse	8 (80)	0	0
Nurse in charge	2 (20)	3 (60)	0
Associate chief nurse	0	2 (40)	0
Associate chief physician	0	0	2 (40)
Chief physician	0	0	3 (60)
**Educational background**			
Undergraduate	10 (100)	0	0
Master	0	5 (100)	1 (20)
Doctor	0	0	4 (80)
**Years of working(Mean ± SD)**	5.00 ​± ​2.62	15.60 ​± ​2.30	14.80 ​± ​7.19
**Years of working in clinical trials(Mean ± SD)**	3.00 ​± ​1.56	9.80 ​± ​1.48	6.60 ​± ​3.05
**Department**			
Department of oncology	3 (30)	1 (20)	1 (20)
Department of endocrinology	1 (10)	0	0
Department of dermatology	1 (10)	1 (20)	1 (20)
Department of neurology	1 (10)	0	0
Phase I clinical trial unit	2 (20)	1 (20)	1 (20)
Department of cardiology	1 (10)	1 (20)	1 (20)
Department of respiratory medicine	1 (10)	1 (20)	1 (20)

CRN, Clinical Research Nurse; NM, Nurse Manager; PI, Principal Investigator; SI, Sub-Investigators.

**Table 3:** Characteristics of CRAs, CRCs and subjects (*N* ​= ​20).

	CRA (*n* ​= ​10) *n* (%)	CRC (*n* ​= ​5) *n* (%)	Subject (*n* ​= ​5) *n* (%)
**Sex**			
Male	3 (30)	0	3 (60)
Female	7 (70)	5 (100)	2 (40)
**Age(Mean ± SD)**	25.70 ​± ​1.49	27.20 ​± ​3.95	30.40 ​± ​6.66
**Educational background**			
Junior college	0	1 (20)	3 (60)
Undergraduate	6 (60)	4 (80)	2 (40)
Master	4 (40)	0	0
**Years of working(Mean ± SD)**	2.90 ​± ​0.99	4.80 ​± ​4.38	0
**Years of working in clinical trials(Mean ± SD)**	2.90 ​± ​0.99	3.40 ​± ​2.61	0

CRC, Clinical Research Coordinator; CRA, Clinical Research Associates.

### The ideal portrait of the professional competence of clinical research nurses

**Fig. 1:**
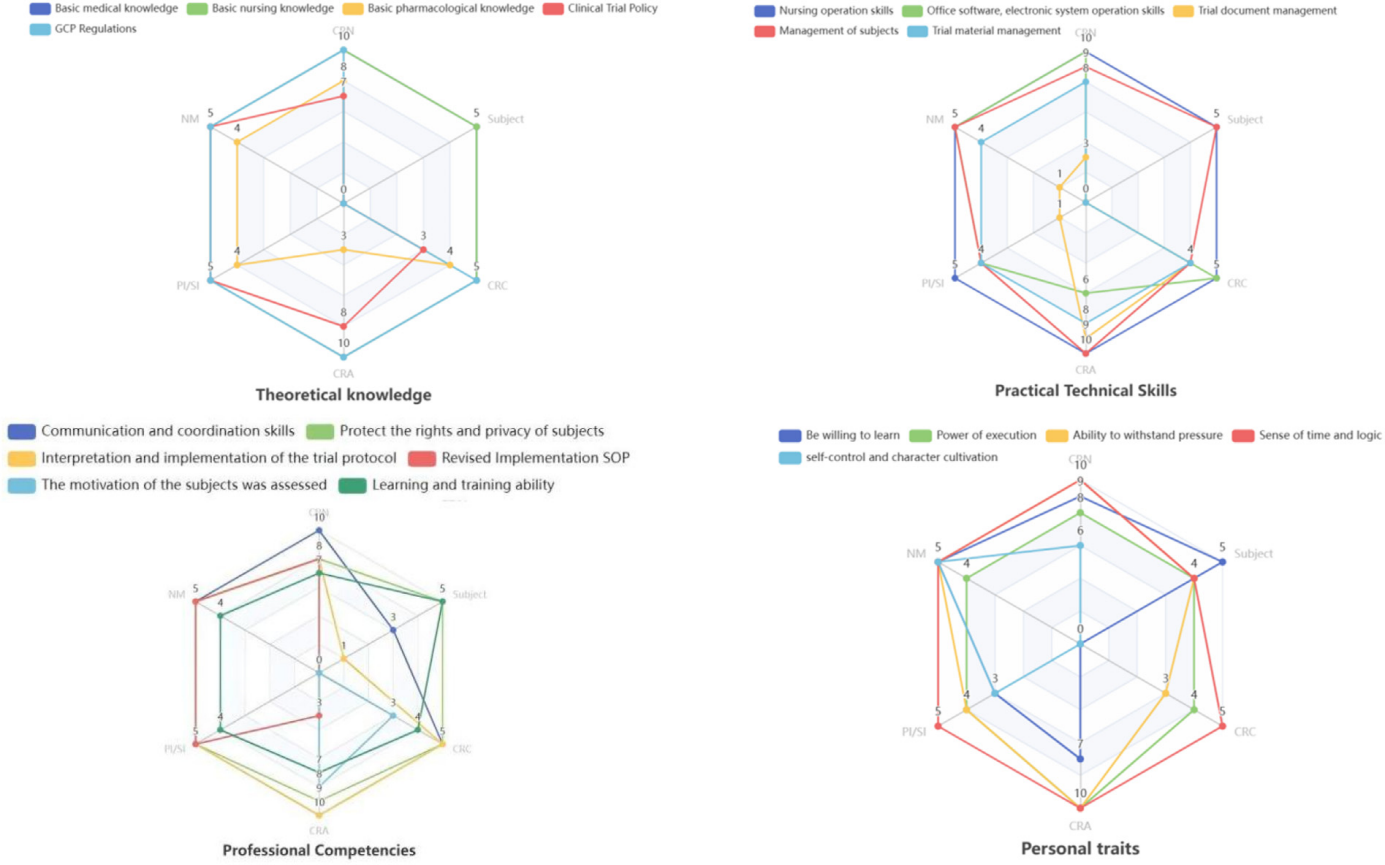
Radar map of word frequency of CRN ideal portrait feature elements. CRN, Clinical Research Nurse.

Based on the interview data, this study identified the ideal profile of a Clinical Research Nurse, categorized into four main themes: theoretical knowledge, practical technical skills, professional competencies, and personal traits. The frequency of themes and their corresponding subcategories is presented in Fig. 1 and Table 4.

**Table 4:** Vocabulary frequency statistics of CRN ideal portrait feature elements.

Items	CRN	NM	PI/SI	CRA	CRC	Subject
**Theoretical knowledge**						
Basic medical knowledge	10	5	5	10	5	5
Basic nursing knowledge	10	5	5	10	5	5
Basic pharmacological knowledge	8	4	4	3	4	0
Clinical trial policy	7	5	5	8	3	0
GCP regulations	10	5	5	10	5	0
**Practical technical skills**						
Nursing operation skills	10	5	5	10	5	5
Office software, electronic system operation skills	10	5	4	6	5	0
Trial document management	3	1	1	9	4	0
Management of subjects	9	5	4	10	4	5
Trial material management	8	4	4	8	4	0
**Professional competencies**						
Communication and coordination skills	10	5	5	10	5	3
Protect the rights and privacy of subjects	8	5	5	9	5	5
Interpretation and implementation of the trial protocol	8	5	5	10	5	1
Revised implementation SOP	8	5	5	3	0	0
The motivation of the subjects was assessed	0	0	0	8	3	0
Learning and training ability	7	4	4	7	4	5
**Personal traits**						
Be willing to learn	9	5	3	7	0	5
Power of execution	8	4	4	10	4	4
Ability to withstand pressure	10	5	4	10	3	4
Sense of time and logic	10	5	5	10	5	4
Self-control and character cultivation	6	5	3	0	0	0

CRN, Clinical Research Nurse; NM, Nurse Manager; PI, Principal Investigator; SI, Sub-Investigators; CRA, Clinical Research Associates; CRC, Clinical Research Coordinator; GCP, Good Clinical Practice.

#### Theme 1: Theoretical knowledge

Both NMs and PIs/SIs emphasized that CRNs should possess comprehensive theoretical knowledge. All respondents consistently noted that basic medical and nursing knowledge is essential for CRNs. Trial subjects were especially concerned about whether CRNs had solid theoretical foundations to ensure their safety and provide appropriate care. Other stakeholders, such as CRAs and CRCs, placed additional demands on CRNs, including a strong awareness of GCP. Strict adherence to GCP guidelines improve trial quality and efficiency. Meanwhile, this study found that CRNs felt they lacked sufficient pharmaceutical knowledge, especially in areas like pharmacokinetics, drug metabolism, and other pharmaceutical-related topics. Given that clinical trials are a core means of drug development, it is vital for clinical trial personnel to possess basic pharmaceutical knowledge. Additionally, NMs and PIs/SIs expressed their expectation that CRNs stay updated with changes in clinical trial policies, both domestically and internationally, and actively learn about evolving requirements. Since trials often span several years, adapting to regulatory changes is crucial for maintaining trial continuity and quality. Participants highlighted specific examples of these competencies:

*PI (C4): "To excel in drug clinical trials, CRNs must have routine medical knowledge. It is also necessary to continually learn about clinical trial regulations and understand policy requirements."*

*Subject (F1): "CRNs must understand my physical condition, know whether I am healthy, and be able to provide immediate care if needed. They need to know my treatment plan and understand both the reasons for and the methods of treatment."*

*NM (B2): "Clinical research nursing is a multidisciplinary role. A qualified CRN should combine the theoretical knowledge of a clinical nurse with concepts specific to clinical research. Typically, CRNs are selected from experienced clinical nurses who have mastered the basics of nursing theory. Thus, they need to focus on expanding their knowledge in clinical trial fields, which includes pharmaceutical theories, ethical knowledge, and regulatory guidelines. The current training for CRNs should be systematically categorized by different knowledge areas and difficulty levels."*

*CRA (D2): "Clinical research nurses are often very busy. They often use their own break time to complete tasks, which can lead to mistakes. It is crucial for CRNs to have a strong GCP awareness, know the trial processes well, and maintain accurate records." For example, mistakes such as missing essential documentation, incorrect data entries, or failing to adhere to scheduled trial procedures could compromise study integrity and regulatory compliance.*

*CRC (E3): "The CRNs I work with are very professional and experienced, especially in tasks like blood collection and examinations. However, their workload can lead to errors, such as missed signatures, incomplete material records, or incorrect details in documentation. CRNs need to pay more attention to GCP requirements to avoid these issues." These documentation errors, if not addressed, may lead to regulatory violations or data inconsistencies, ultimately affecting trial validity.*

*CRN (A6): "When our department first started clinical trials, we were asked to complete GCP training before taking on projects. Despite feeling uncertain at first, we have now managed 5 to 6 projects over the past two years. Since then, our department has also implemented additional training sessions and lectures to improve our capabilities."*

These findings illustrate the need for structured and systematic training in areas such as GCP compliance, pharmaceutical knowledge, and effective management of trial processes. By focusing on these areas, CRNs can enhance their ability to contribute effectively to clinical trials, ensuring both participant safety and trial success.

#### Theme 2: Practical technical skills

All respondents agreed that CRNs should possess strong nursing operational skills, with nearly all emphasizing the importance of effective subject management. It is noteworthy that clinical trials have gradually transitioned into the digital era, with the incorporation of advanced technologies and high-tech equipment in many clinical trial institutions. This shift has heightened the need for CRNs to develop robust office and system operation skills. Additionally, CRAs and CRCs pointed out that the ability of CRNs to manage subjects, trial materials, and documents varies significantly. We also found that responsibilities for trial document and material management often involved multiple roles, leading to unclear boundaries of responsibility. Due to their demanding clinical duties, CRNs often struggle to dedicate sufficient time to managing documents and materials, and a considerable portion of these tasks is taken on by CRCs. Therefore, CRNs, along with other team members, need to manage the materials and documents under their purview effectively, while also learning to facilitate smooth handovers and collaboration. Early in the trial process, it is crucial to establish well-defined responsibilities for all team members. Participants provided specific insights:

*Subject (F5): "This is my first time participating in a clinical trial, and I need instructions on when and how to do things. Although I was informed about the overall process at the beginning, it was difficult to remember all the details. The clinical research nurse at this center has been very diligent in guiding me through each step, which helps put me at ease."*

*CRN (A8): "Clinical research nurses need to effectively manage subjects during the trial, providing thorough screening and education during the early stages. During the trial, we address subjects' questions to improve compliance, and arrange follow-ups after the trial. Additionally, careful scheduling is essential—some subjects coming in for examinations may have adverse events or fail to meet examination requirements, and since these subjects are not hospitalized, they need guidance on when and where to come for follow-up visits."*

*NM (B1): "Nursing operational skills are a fundamental requirement. Our department holds weekly summary meetings and monthly learning sessions, where senior nurses are tasked with training others. While traditional nursing operations are well-covered, new technologies require additional training. For example, this year we worked with a needle-free insulin pump, which had excellent results but was quite complicated to operate. Incorrect use could cause skin injuries, so it was essential for nurses to learn the process thoroughly before use."*

*CRA (D5): "In monitoring clinical trials, effective subject management by CRNs not only improves compliance and ensures the steady progress of the trial but is also crucial for collecting complete experimental data and improving trial quality."*

*CRC (E4): "During clinical trials, numerous documents are generated, involving research doctors, CRNs, quality control personnel, and subjects. Many of these documents are stored in multiple locations, resulting in overlapping management responsibilities. In practice, we often handle the sorting and filing of documents early in the process. When documents need modifications or additions, we use post-it notes to prompt the relevant individuals."*

These findings highlight the need for CRNs to adapt to evolving technology in clinical trials, enhance their operational and subject management skills, and work effectively in collaboration with others involved in the trial. Standardizing responsibilities and clearly defining roles will help ensure smoother processes and improved trial outcomes.

#### Theme 3: Professional competencies

Almost all respondents agreed that CRNs need strong communication and coordination skills, the ability to protect subjects’ rights and interests, and a commitment to continuous learning and training. CRAs and CRCs emphasized the importance of CRNs being able to interpret and implement trial protocols effectively. CRAs also noted that assessing trial subjects' motivations could improve data integrity and trial continuity. Although trial subject inclusion and exclusion criteria are strictly followed according to the protocol, a substantial number of trial subject withdraw from trials for reasons unrelated to medical concerns. The NMs and PIs/SIs interviewed expressed concern regarding CRNs' ability to revise and implement Standard Operating Procedures (SOPs). Conducting trial operations in strict accordance with SOPs is fundamental to ensuring trial quality and data traceability. Different types of clinical trials require SOPs tailored to specific needs, and managers hope to see CRNs actively contributing to the improvement and development of SOPs through their practical experience, thus broadening the types of trials conducted within their departments. Participants shared specific insights:

*CRN (A5): "One of my key responsibilities is ensuring that subjects fully understand the informed consent process. Many patients, especially elderly individuals or those with limited education, struggle to comprehend complex medical terminology. I often spend extra time simplifying explanations and confirming their understanding. Additionally, protecting patient confidentiality is crucial, particularly when handling sensitive trial data. Ethical dilemmas arise when family members attempt to influence subjects' decisions, and in such cases, I must carefully balance respecting the participant’s autonomy while maintaining open communication with their family. To navigate these challenges, I rely on continuous ethics training and discussions with the research team to uphold both patient rights and trial integrity."*

*PI (C3): "CRNs need to communicate and coordinate with different roles throughout the trial process. Efficient communication and collaboration are crucial for the steady progress of trials. All operations must strictly follow the SOP, but as trials develop and iterate, there are sometimes lags in SOP updates. Operators need to self-check and improve the SOPs they use."*

*CRA (D6): "The project initiation meeting explains the process in detail, but since trials often last for a long time and CRNs are busy, they may need reminders. Understanding why subjects participate in clinical trials and recognizing their social roles can help reduce dropout rates. In one project I managed, many subjects withdrew not due to adverse drug reactions but because of scheduling conflicts, such as students returning to school or hourly workers unable to align work schedules with trial timelines."*

*CRC (E2): "When working with CRNs, we clearly explain each step and process. Familiarity with the trial process by all authorized nurses is critical for smooth implementation. I understand the pressure CRNs face, as many handle multiple projects while also caring for patients, so I try to be clear about each step, including instructions on recording and signing."*

*Subject (F2): "I joined the trial to earn some money, but I want my personal information kept confidential, and I don't want my family to know about it."*

*NM (B3): "Recently, our department managed an external medicine trial, which we successfully completed thanks to continuous learning. We held an internal training session where nurses shared knowledge from the trial. The nursing procedures must strictly adhere to SOPs, but when the current SOPs are incomplete, CRNs are responsible for revising them. Our SOPs are updated annually, and CRNs are expected to report any issues and make necessary revisions promptly."*

These findings underscore the importance of comprehensive training in communication, coordination, and protocol adherence for CRNs. Their ability to interpret trial protocols, manage SOPs, and effectively communicate with other trial subjects is essential for ensuring trial quality and the safety and rights of the subjects. Regularly updating SOPs and involving CRNs in the revision process can lead to better trial outcomes and improved team efficiency.

#### Theme 4: Personal traits

More than 80% of the respondents highlighted that CRNs should possess strong execution skills, stress tolerance, and logical thinking. The interviewed NMs and PIs/SIs emphasized that CRNs need to continuously learn from practice, analyzing and summarizing the differences and similarities among various clinical trials. A key challenge for CRNs is balancing clinical nursing duties with research responsibilities, which requires not only stress management but also effective communication and role adaptation. Clinical trials come in many forms, with increasingly diverse types—from early chemical drugs to biologics, vaccines, and diagnostic reagents. Each trial presents new protocol requirements, timelines, and operational challenges, requiring CRNs to adapt quickly while maintaining strict adherence to research protocols. Most respondents noted that a key distinction between clinical trials and routine nursing is the rigorous time-sensitive nature of research procedures. To support CRNs in managing these dual roles, interdisciplinary collaboration, structured training programs, and clear role delineation within research teams are essential strategies. In clinical trials, procedures often require precise timing down to the second, making time management and logical reasoning essential traits for CRNs. Specific insights were shared by participants:

*CRA (D9): "I believe a good clinical research nurse should be able to advance the trial process according to the protocol and perform all procedures as required by the SOP."*

*NM (B1): "Our department is involved in various projects, including oral drugs, topical medications, injectables, and medical devices. Each trial is quite different, and while we can learn from past experiences, many trials require learning new skills from scratch, which can be challenging for CRNs. To address this, we have each team member report and share their experiences as a form of training. This helps build practical experience, making it easier to handle similar projects in the future."*

*CRN (A7): "The CRNs I work with, including myself, are also involved in clinical nursing within the department. This means we must constantly switch between two different types of work and manage the pressures of both clinical and trial roles. Since beginning clinical trial work, I’ve found it challenging to keep up. Unlike daily nursing tasks, clinical trials have strict time requirements. Every procedure has a specific time window, and these windows often differ, which takes considerable effort to understand and remember."*

These findings illustrate that CRNs must not only have solid operational skills but also be able to manage the complexities of different trials and adapt quickly. Their ability to handle strict time constraints and maintain logical thinking, coupled with strong stress tolerance, is critical for ensuring the smooth progress of clinical trials. To cope with work-related stress, CRNs commonly use self-regulation techniques such as mindfulness and deep breathing. Institutional support, including peer support programs and psychological counseling, has been shown to reduce burnout. Additionally, effective time management strategies, such as task prioritization, help CRNs balance workloads. Integrating stress management training into CRN professional development programs may further enhance their resilience and job performance.^19^ Continuous learning, knowledge exchange, and practical experience are key to their professional growth in this demanding field.

## Discussion

### Diversification of CRN's ideal professional competence profile

This study conducted interviews with various clinical trial participants, from hospitals to enterprises, sponsors to contractors, and operators to managers, to understand the expectations of each role in clinical trials regarding CRN competencies. From these different perspectives, an ideal profile of CRN professional abilities was developed. This profile includes four primary competency categories and 21 secondary indicators, highlighting the diverse characteristics of the role. These insights provide a foundation for building a CRN training system and help clarify expectations from different stakeholders, improving collaboration and efficiency.

All six participant groups agreed that fundamental medical knowledge, nursing skills, ethical oversight, and operational expertise form the core of CRN professional competencies. This finding underscores that CRN training builds upon the foundation of clinical nursing education. In 2016, the American Nurses Association recognized clinical research nursing as a specialized practice, emphasizing that CRNs are expert nurses who possess deep theoretical knowledge and advanced clinical skills.^1^ While CRNs are not formally classified as specialist nurses in many countries, their role involves a high degree of specialization in clinical trials. Similar to specialist nurses, such as stoma nurses and diabetes educators, have long played pivotal roles in their respective fields.^20^, ^21^, ^22^ This recognition highlights the growing need for structured training and competency frameworks to support CRN professional development. Previous studies have highlighted that although CRNs possess clinical nursing experience, the content and frequency of their training in nursing knowledge and skills vary significantly across different departments.^5^ The expectations regarding specialized nursing knowledge also differ widely depending on the area of clinical research. For instance, CRNs involved in oncology projects and external medicine trials are required to master nursing theories and skills specific to their respective fields.^23^ Beyond specialized medical knowledge, CRNs also play a vital role in ethical oversight and protecting the rights and privacy of subjects. As the primary point of contact between trial subjects and the research team, CRNs are responsible for ensuring that subjects fully understand the study's objectives, potential risks, and benefits. In clinical trials, especially those involving vulnerable populations, inadequate comprehension of informed consent documents can lead to ethical violations and reduced patient compliance. Studies have shown that CRNs often face challenges in managing informed consent in cases where subjects have limited health literacy or where family influence affects decision-making.^24^ Additionally, issues such as confidentiality protection, ethical decision-making in adverse events, and balancing research and patient care responsibilities require more in-depth ethical training.^25^ This study also found that CRNs need to pay greater attention to the mental health and motivations of trial subjects compared to clinical nurses. The primary motivation for most subjects in phase I clinical trials is to receive financial compensation, and many “professional subjects” make a living by participating in these trials. These individuals are often challenging to manage, sometimes even feigning discomfort to obtain higher compensation. In contrast, subjects in phase II, III, and IV clinical trials are typically patients seeking alternative treatments because existing drugs are ineffective or unaffordable. Some subjects may hide their involvement from family members and often experience feelings of pessimism, anxiety, and depression. Effective communication and emotional support from CRNs can help these subjects undergo treatment smoothly, ensure trial continuity, and prevent irreversible psychological harm. To enhance CRN ethical competency, it is recommended that future training programs incorporate case-based ethical discussions, scenario simulations, and interdisciplinary workshops involving bioethicists, regulatory experts, and experienced clinical investigators. These approaches could better prepare CRNs for the ethical complexities they encounter in practice, ultimately improving participant protection and trial integrity. Thus, in addition to clinical and technical training, CRN development programs should incorporate research ethics education, communication strategies for informed consent, and standardized protocols for addressing ethical dilemmas. A more structured, comprehensive, and ongoing ethics training system is necessary to ensure that CRNs are fully equipped to handle the ethical challenges in clinical trials.

There are distinct priorities between trial subjects and implementers (CRNs, NMs, PIs/SIs, CRAs, and CRCs). Despite all participants highlights CRNs' role in the informed consent process, ensuring subject understanding, ethical compliance, and psychological support, subjects are more concerned about whether CRNs could manage their time efficiently and safeguard their rights and privacy. In contrast, implementers were more focused on CRNs' knowledge of GCP regulations, document management, and team coordination.^26^ As is known to us, the primary goal of clinical trials is to protect the health and rights of subjects while improving their compliance and ensuring they complete the entire trial process.^27^ It is worth mentioning that similar to previous studies, our study revealed that subjects often lacked knowledge about the trial procedures and CRN responsibilities.^28^ Therefore, it is recommended that CRNs enhance education and communication efforts with subjects to boost their sense of involvement, which wil support the quality of the CRN's work. This study found that trial subjects valued the guidance provided by CRNs. In Phase I trials, some suggested adding recreational activities, but managers emphasized the need for strict inpatient monitoring. CRNs must balance participant comfort with protocol adherence. In Phase II-IV trials, CRNs play a key role in coordinating follow-ups and guiding subjects through medical procedures, ensuring adherence to protocols while enhancing the trial experience. Efficient guidance throughout these processes can greatly reduce waiting times, improve subject experience, enhance compliance, and help ensure the smooth progress of clinical trials.

Meanwhile, both managers (NMs, PIs/SIs) and operators (CRNs) had more comprehensive requirements for CRNs. We found that all managers emphasized the importance of CRNs having basic pharmacological knowledge and staying up to date on changes in clinical trial policies. Similar to previous studies, this study also found that most CRNs lacked sufficient pharmacological knowledge, preventing them from thoroughly understanding trial protocols and potentially affecting trial quality.^29^, ^30^, ^31^ Pharmacological understanding is crucial for CRNs, as they play a key role in drug administration, monitoring adverse reactions, and ensuring protocol adherence. Additionally, insufficient pharmacological knowledge may hinder CRNs’ ability to detect and report adverse events (AEs) accurately. Research has indicated that underreporting or misclassification of AEs in clinical trials can compromise patient safety and lead to biased trial results, ultimately affecting the regulatory approval process of new drugs.^32^ Furthermore, lack of familiarity with trial-specific medications may impact CRNs' ability to educate trial subjects effectively, potentially reducing adherence to treatment regimens and increasing dropout rates. To mitigate these risks, it is recommended that CRNs receive systematic pharmacological training, possibly facilitated by hospital pharmacy departments. Additionally, CRNs should regularly attend Q&A sessions and relevant lectures. With the introduction of new versions of GCP guidelines, regulatory agencies have imposed clearer requirements for clinical trials.^33^, ^34^, ^35^ To ensure compliance, it is crucial for CRNs and all trial subjects to stay informed on these updates. Therefore, it is suggested that relevant departments follow policy changes closely and offer timely training and lectures. It is worth noting that this study found that several managers highlighted the importance of participant feedback and evaluation as key indicators for assessing CRN performance. Subjects, as the primary participants in clinical trials, should be treated as individuals, not merely as “experimental subjects.” Collecting feedback from subjects, who are in close contact with CRNs, can significantly enhance CRN development by fostering effective communication, guiding improvements, and building trust—ultimately contributing to the successful progress of clinical trials. Notably, some institutions involved in the study collect feedback scores from subjects at different stages—early, mid, and before completing their participation in clinical trials. These scores are periodically reported and used to implement corrective measures. Ethical safety considerations for trial subjects have evolved beyond the fundamental rights of life safety, privacy, financial compensation, and informed consent. Subjects deserve respect, recognition, and social and familial honor, which should be ensured by all parties involved in the trial to create a comprehensive protective environment. Collecting participant feedback and evaluations of CRNs is an essential step for managers in emphasizing the ethical aspects of clinical trials.

Moreover, there are notable differences between hospital-based participants (CRNs, NMs, PIs/SIs) and enterprise-based participants (CRCs, CRAs). Our study showed that sponsors placed greater emphasis on assessing subjects' social roles and motivations, as well as on managing trial materials and documents. While protocols specify acceptable dropout rates, funders prefer stable subjects to ensure smooth processes and consistent outcomes. It is noteworthy that we found Site Management Organizations (SMOs) and Contract Research Organizations (CROs) strive to include as many subjects as possible, provided they meet the trial protocol requirements. This approach may be linked to the fact that the number of subjects who complete the trial directly impacts the amount of contract payments. Consistent with previous studies, comprehensive documentation and systematic classification of trial records are essential.^36^, ^37^, ^38^ Timely documentation and management are crucial for effective coordination and monitoring by CRAs and CRCs. Importantly, managing trial process documentation requires collaboration from all parties involved. However, our findings indicate that, compared to enterprise personnel, hospital staff tend to place less emphasis on managing trial process documents, leaving much of this responsibility to CRAs or CRCs. In light of this, it is recommended that CRNs not only evaluate subjects’ motivation and social roles more thoroughly but also improve their document and material management. Establishing SOPs for material and document traceability could further enhance trial efficiency and quality.

### Comparison with international CRN competency frameworks

The findings of this study align with international CRN competency frameworks, such as the Oncology Nursing Society (ONS) Clinical Research Nurse Competency Framework (2016).^13^ The ONS framework emphasizes core competencies in scientific knowledge, patient care coordination, protocol adherence, and regulatory compliance, which are consistent with our identified themes of theoretical knowledge, practical skills, professional competencies, and personal traits.

However, our study highlights some context-specific differences. For example, while the ONS framework underscores advanced pharmacological knowledge as a key competency, our findings indicate that many CRNs in China lack sufficient pharmacological training, potentially affecting trial quality. Additionally, stress management and workload distribution were frequently mentioned by our participants, suggesting a need for stronger psychological support systems for CRNs in China. Unlike in some Western countries where CRNs function as independent research professionals, Chinese CRNs often juggle both clinical nursing duties and research responsibilities, leading to increased workload and stress. Additionally, regulatory policy updates pose challenges unique to China, as CRNs must frequently adapt to evolving clinical trial requirements with limited standardized training resources. Moreover, team collaboration dynamics in China differ from international settings. CRNs here often work closely with principal investigators, coordinators, and research sponsors, yet their professional identity and role boundaries remain less clearly defined. This underscores the need for a more structured competency framework and role clarification in the Chinese health care system. Addressing these unique challenges could help strengthen CRN training programs and improve clinical trial quality.

These comparisons suggest that while CRN competencies share common global standards, localized training programs should address specific challenges faced by CRNs in China.

### Adapting Western CRN training models to the Chinese context

Western countries, particularly the United States and the United Kingdom, have well-established CRN training frameworks, such as the training programs developed by the National Institutes of Health (NIH), the Oncology Nursing Society (ONS), and the UK's National Institute for Health Research (NIHR).^13^^,^^39^^,^^40^ These programs emphasize standardized pharmacological education, ethical compliance, and independent research management skills, which could serve as valuable references for CRN training in China.

However, due to differences in health care structures and clinical trial environments, directly adopting Western models may not be feasible. In China, CRNs often balance both clinical nursing and research responsibilities, making it essential to develop integrated training programs that allow for flexible learning while addressing the heavy workload. Another key area for adaptation is the regulatory landscape. Unlike Western countries with long-established research nurse career pathways, China's CRN role is still evolving. Establishing national certification programs and clear role definitions—similar to Western credentialing systems—would help standardize training while allowing flexibility for local health care settings.

### Practical and comprehensive profile of CRN professional competence

The successful execution of clinical trials depends on multidisciplinary collaboration, with CRNs playing a critical and indispensable role.^41^^,^^42^ By collecting and analyzing the ideal CRN professional competence profiles from different trial subjects, this study provides a more comprehensive understanding of the required skills. It offers valuable insights for shaping CRN professional characteristics and improving the reliability of qualitative research. This study identified four major competency categories and 21 specific indicators for CRNs, providing a solid reference for developing a more robust CRN training system.

### Strengths and limitations

This study employed phenomenological methods to analyze the expectations of various roles and stakeholders involved in clinical trials regarding clinical research nurses. By considering perspectives from management, CRNs themselves, hospitals, and enterprises, the study depicted the ideal profile of CRNs as envisioned by clinical trial subjects. We explored the key elements of CRN professional competencies from multiple dimensions, including the quality of clinical trials, the physical and mental needs of subjects, and the development of CRNs.

Sample limitations, such as not covering private hospitals or different levels of care, may affect the generalizability of our findings. Future studies can expand the sample scope to include other provinces and hospitals at different levels (including private hospitals, secondary hospitals, etc.) for comparative analysis, so as to further verify the universality of CRN capability characteristics.

## Conclusions

This study has preliminarily explored the key elements of professional competence that CRNs in China should possess, identifying four core themes: theoretical knowledge, practical technical skills, professional quality, and personal characteristics. These competencies were identified based on the expectations and perceptions of various clinical trial stakeholders, including PIs, NMs, CRAs, CRCs, and trial subjects. Their insights provide a valuable perspective on the competencies CRNs should develop to meet the demands of clinical research. Looking ahead, the research team plans to employ the Delphi method to construct a professional competency evaluation model for CRNs. By conducting expert consultations and reliability and validity studies, the team aims to develop a quantifiable evaluation scale that reflects both stakeholder expectations and real-world competency requirements, ultimately creating targeted training programs to ensure CRN training is more scientific and practical.

## CRediT authorship contribution statement

**Heng Yang:** Conceptualization, Methodology, Data collection, Formal Analysis, Writing – original draft, Writing – review and editing. **Yipei Chen:** Supervision, Writing – review and editing. **Xin Peng:** Conceptualization and Methodology, Project administration. All authors approved the final version for submission.

## Ethics statement

This study was approved by the Medical Ethics Committee of Tongji Medical College, Huazhong University of Science and Technology (Approval No. 2024-S153, 24 July 2024). Informed consent to participate was obtained from all participants prior to the enrollment of this study.

## Data availability statement

The data that support the findings of this study are available from the corresponding author, XP upon reasonable request.

## Declaration of generative AI and AI-assisted technologies in the writing process

No AI tools/services were used during the preparation of this work.

## Funding

This study received no external funding.

## Declaration of competing interest

The authors declare no conflict of interest.
