# Supplementary material for: An ideal portrait of the professional competence of clinical research nurses: A qualitative study
Source: Asia Pac J Oncol Nurs. 2025 Mar 8;12:100682. doi: 10.1016/j.apjon.2025.100682 (PMC11976228; doi:10.1016/j.apjon.2025.100682)
Supplement: Multimedia component 1 [file mmc1.docx]

##### ****Example of Coding Process****

| **Participant Statement (Verbatim)** | **Initial Code** | **Subcategory** | **Theme** |
| --- | --- | --- | --- |
| "CRNs must be familiar with medical knowledge and understand drug mechanisms to ensure patient safety in trials." | Medical knowledge, drug mechanisms | Basic medical knowledge, Basic pharmacological knowledge | Theoretical Knowledge |
| "I often feel overwhelmed by the number of documents and reports I need to manage in each trial." | Workload, document management | Ability to withstand pressure, Trial material management | Personal Traits, Practical Technical Skills |
| "CRNs should maintain a calm attitude when dealing with stressed participants and provide psychological support." | Emotional support | Communication | Professional Competencies |
| "I find that having a strict schedule and checklists helps me stay organized and on track during trials." | Time management, organizational skills | Sense of time and logic, self-control and character cultivation | Personal Traits |
